# Microbiome and metabolome modifying effects of several cardiovascular disease interventions in apo-E^−/−^ mice

**DOI:** 10.1186/s40168-017-0246-x

**Published:** 2017-03-13

**Authors:** Paul M. Ryan, Lis E. E. London, Trent C. Bjorndahl, Rupasri Mandal, Kiera Murphy, Gerald F. Fitzgerald, Fergus Shanahan, R. Paul Ross, David S. Wishart, Noel M. Caplice, Catherine Stanton

**Affiliations:** 1 Department of Food Biosciences, Teagasc Food Research Centre, Moorepark, Fermoy, Co. Cork, Ireland; 2 School of Microbiology, University College Cork, Co. Cork, Ireland; 3 Department of Biological Sciences, University of Alberta, Edmonton, AB Canada; 4 Department of Medicine, University College Cork, National University of Ireland, Cork, Ireland; 5 APC Microbiome Institute, Biosciences Institute, University College Cork, Co. Cork, Ireland; 6 College of Science, Engineering & Food Science, University College Cork, Co. Cork, Ireland; 7 Department of Computing Science, University of Alberta, Edmonton, AB Canada; 8 National Institute for Nanotechnology, Edmonton, AB Canada; 9 Centre for Research in Vascular Biology, University College Cork, Co. Cork, Ireland

**Keywords:** Apo-E-deficient, Atherosclerosis, Cardiovascular disease, Microbiome, Metabolome

## Abstract

**Background:**

There is strong evidence indicating that gut microbiota have the potential to modify, or be modified by the drugs and nutritional interventions that we rely upon. This study aims to characterize the compositional and functional effects of several nutritional, neutraceutical, and pharmaceutical cardiovascular disease interventions on the gut microbiome, through metagenomic and metabolomic approaches. Apolipoprotein-E-deficient mice were fed for 24 weeks either high-fat/cholesterol diet alone (control, HFC) or high-fat/cholesterol in conjunction with one of three dietary interventions, as follows: plant sterol ester (PSE), oat β-glucan (OBG) and bile salt hydrolase-active *Lactobacillus reuteri* APC 2587 (BSH), or the drug atorvastatin (STAT). The gut microbiome composition was then investigated, in addition to the host fecal and serum metabolome.

**Results:**

We observed major shifts in the composition of the gut microbiome of PSE mice, while OBG and BSH mice displayed more modest fluctuations, and STAT showed relatively few alterations. Interestingly, these compositional effects imparted by PSE were coupled with an increase in acetate and reduction in isovalerate (*p* < 0.05), while OBG promoted n-butyrate synthesis (*p* < 0.01). In addition, PSE significantly dampened the microbial production of the proatherogenic precursor compound, trimethylamine (*p* < 0.05), attenuated cholesterol accumulation, and nearly abolished atherogenesis in the model (*p* < 0.05). However, PSE supplementation produced the heaviest mice with the greatest degree of adiposity (*p* < 0.05). Finally, PSE, OBG, and STAT all appeared to have considerable impact on the host serum metabolome, including alterations in several acylcarnitines previously associated with a state of metabolic dysfunction (*p* < 0.05).

**Conclusions:**

We observed functional alterations in microbial and host-derived metabolites, which may have important implications for systemic metabolic health, suggesting that cardiovascular disease interventions may have a significant impact on the microbiome composition and functionality. This study indicates that the gut microbiome-modifying effects of novel therapeutics should be considered, in addition to the direct host effects.

**Electronic supplementary material:**

The online version of this article (doi:10.1186/s40168-017-0246-x) contains supplementary material, which is available to authorized users.

## Background

Cardiovascular diseases represent the most fatal maladies of the system-wide metabolic dysfunction observed in millions globally, currently responsible for an estimated ~30% of mortalities each year [1]. Circulating low-density lipoprotein cholesterol (LDL-C) and high-density lipoprotein cholesterol (HDL-C) levels are key modifiable markers in atherosclerosis development and, as a result, these have been the targets of the vast majority of nutritional and pharmaceutical cardiovascular disease interventions. Although external factors such as physical exercise and diet play primary roles in cardiovascular disease onset, it has become evident that the trillions of microorganisms indigenous to our gut also contribute [2–4]. These bacteria and other microbes express a diverse array of genes, many of which play important roles in metabolic function and dysfunction. Such communities respond directly to the nutritional and pharmaceutical environment that their host provides them with, and this largely dictates important host metabolic characteristics such as hormonal and inflammatory tones [5, 6]. While some of these effects can have beneficial outcomes for host metabolic health, the promotion of certain species has even been correlated with cardiovascular disease pathogenesis (for review [7]).

However, we are now discovering that these microbes can express enzymes capable of interacting and interfering with the nutritional and pharmaceutical interventions which we consume, ultimately impacting on their bioactivity. There is growing appreciation for the gut microbiome modulating capacity of many functional nutritional ingredients, such as phytosterols [8] and non-digestible polysaccharides [9, 10], yet this still stems from a limited number of studies for many ingredients. On the other hand, a review reportedly identified 30 common drugs which were open to metabolism by bacterial enzymes, through proteolysis, reduction, dehydroxylation, hydrolysis, and other reactions [11]. The authors predicted that the number of drug-microbiota interactions identified would increase rapidly in the coming years. One drug which appears to act at least in part through the microbiota is metformin, a widely used therapeutic for metabolic dysfunction. It was initially noted that the effects of metformin were compromised by the application of certain antibiotics; however, more recent studies have demonstrated an enrichment of *Akkermansia muciniphila* in the microbiota of metformin-treated animals [12], and the corrective effects of *Akkermansia muciniphila* in models of metabolic dysfunction have previously been demonstrated by Everard et al. [13]. Statins, as one of the most widely consumed drug families globally, are of considerable interest in this regard.

In addition, several functional food therapeutics have gained high-quality scientific support in recent years. Current meta-analyses demonstrate that oat β-glucan dietary supplementation consistently reduces LDL-C, non-HDL, and apolipoprotein (apo)-B particles in moderately hyperlipidaemic individuals [14, 15]. While the reductions are somewhat modest, oat β-glucan supplementation may represent an effective and safe method for the management of cardiovascular disease risk. Indeed, it is likely that such functional food ingredients act through manipulation of the indigenous microbiome [16]. Another interventional design currently being explored is the direct application of bacteria expressing an enzyme of interest, such as the bile salt hydrolase family [17, 18]. One such organism, which has reached the market following several successful randomized clinical trials, is *Lactobacillus reuteri* NCIMB 30242. The strain has demonstrated the potential to reduce LDL-C, non-LDL-C, and apo-B100 [19], as well as several inflammatory markers associated with atherogenesis [20].

The mechanisms of action of widely used functional foods, and even certain prescribed pharmaceutics, have traditionally often remained partially unclear. It is likely that many of these interventions act indirectly on their intended target by modifying the host gut microbiota, or being modified by the gut microbiota. It may be important to elucidate these microbial interactions in order to personalize patient therapies. In the present study, we used the apo-E^−/−^ murine model which closely mirrors the pathophysiology of human atherosclerosis [21]. Apo-E is essential to the recognition of esterified cholesterol-rich particles and subsequent cholesterol uptake by hepatocytes. Without this cycle, an atherogenic lipid profile will develop and prevail, along with atherosclerotic plaque formation. The ultimate purpose of this study was to characterize the compositional and functional alterations to the microbiome and metabolome following courses of commercially available nutritional and pharmaceutical cardiovascular disease interventions.

## Methods

### Animals and diets

Four-week-old male ApoE^tm1Unc/J^ mice were acquired from the Jackson Laboratory (JAX, through Charles River Laboratories International), housed under barrier-maintained conditions within the Biological Service Unit, University College Cork. Animals were acclimatized for 2 weeks prior to being randomized into six separate groups (A-E *n* = 14, F *n* = 7). The four intervention groups were as follows: (A) a bile salt hydrolase-active probiotic *Lactobacillus reuteri* APC 2587 (BSH; 10^9^ CFU/day), (B) plant sterol ester (PSE; 3.4% *w*/*w*; Raisio), (C) oat β-glucan (OBG; 7% *w*/*w*; OatWell), and (D) atorvastatin (STAT; 1 · 5 mg/kg). All intervention groups received a high-fat/cholesterol (HFC; 42% fat, 1.25% cholesterol; Harlan) diet alongside treatment for a 24-week period. In addition, (E) HFC diet control and (F) normal chow control (NC) were run concurrently.

### Bacteria and culturing

A rifampicin-resistant derivative of BSH was created and stored in 70% *v*/*v* glycerol, at −80 °C. From these stocks, bacteria were routinely cultured anaerobically at 37 °C on de Man Rugosa Sharp (MRS; Difco; 1 · 5% *w*/*w* agar) with the addition of 100 μg/ml *w*/*v* rifampicin. From these plates, single colonies were inoculated in 5 ml MRS-rifampicin broth and grown overnight under the same conditions. Bacteria were subcultured twice more prior to freeze-drying.

Bacteria were cultured as above until stationary phase and centrifuged (16,900 × *g* for 15 min, at 4 °C; SLA-3000 rotor, Sorvall RC B5-Plus). The resulting cell pellet was washed twice with phosphate buffered saline (Sigma Aldrich). Cells were then resuspended at ~2 × 10^10^ CFU/ml in sterile 15% (*w*/*v*) trehalose (Sigma Aldrich), and 1 ml aliquots were dispensed into sterile 2 ml lyophilisation vials. The vials were lyophilized on a 24-hour program (freeze temperature −40 °C, additional freeze 1 min, condenser set point −60, vacuum set point 600 mTorr; VirTis AdVantage Wizard 2·0) and stored at 4 °C until use. Bacteria were resuspended in distilled water each day in order to deliver 10^9^ CFU/day to each animal. Gastric transit of the strains was assessed each week by culturing serial dilutions of fresh fecal samples on MRS-rifampicin plates, as described previously.

### Biochemical and biometric analysis

At several points during intervention, mice were bled by facial vein puncture. Whole blood was allowed to clot on ice for 30 min prior to centrifugation at 1000 × *g* for 10 min at 4 °C. Serum was withdrawn and stored at −80 °C until use. Total serum cholesterol levels were determined in duplicate as per manufacturer’s instructions using the EnzyChrom colorimetric assay (ECCH-100, BioAssay Systems, Hayward, CA, USA). Serum HDL-C, LDL-C, and triglycerides (TG) were determined by their respective LabAssay assays (Wako Diagnostics). Fecal and liver lipids were extracted from approximately 50 mg of sample with a 2:1 (*v*:*v*) mixture of chloroform:methanol according to the method of Folch et al. [22]. The resulting lipids were dried and resuspended in the appropriate buffer prior to assay. The same kits used for serum lipid analysis were used for analysis of the extracts.

Intestinal alkaline phosphatase (IAP) activity was assessed in protein extracts from 50 mg of jejunum tissue using the SensoLyte pNPP Alkaline Phosphatase Assay Kit (AnaSpec, Fremont, CA USA) following the manufacturer’s instructions. Data are expressed as microgram of pNPP hydrolyzed per minute per milligram of protein.

Following the 24-week intervention, all mice were analyzed in a Minispec mq benchtop NMR spectrometer (Bruker Instruments) for fat and lean body mass. Animals were then culled and tissues collected and weighed.

### Atherosclerotic plaque scoring

Atherosclerotic lesions were quantified by en face analysis of the whole aorta. For the en face analysis of the aorta, the aorta were paraformaldehyde (PFA) fixed (4% *w*/*v*) the Oil-Red-O-stained aorta were photographed and used for the quantification of atherosclerotic lesions [23]. The total aortic surface area and the lesion area were measured by image analysis (NIH ImageJ; version 1.49), and the ratio of the lesion area to the total area was calculated.

### Cecum microbiome

Microbial DNA was extracted from cecal content using the QIAamp DNA Stool Mini Kit protocol (Qiagen), adapted to include a bead-beating step. The V3-V4 regions of the 16S gene were amplified using the primer pair 5-TCGT CGGC AGCG TCAG ATGT GTAT AAGA GACA GCCT ACGG GNGG CWGC AG-3 and 5-GTCT CGTG GGCT CGGA GATG TGTA TAAG AGAC AGGA CTAC HVGG GTAT CTAA TCC-3, as per the preparation instructions for Illumina MiSeq. Samples were barcoded using the primer combinations available in the Illumina Nextera kit (Illumina Nextera XT, Illumina Sweden) prior to quantification with the Qubit High Sensitivity DNA kit (Life Technologies), equimolar pooling and high-throughput sequencing on the MiSeq platform.

FLASH (FLASH: fast length adjustment of short reads to improve genome assemblies) was applied for the assembly of resulting 300-bp paired-end reads. Additional sequence read processing, which included quality filtering based on a quality score of >25 and removal of mismatched barcodes and sequences below length thresholds, was performed within QIIME (version 1.8.0). USEARCH (version 7, 64-bit) was utilized for denoising, chimera detection, and clustering into operational taxonomic units (OTUs) (97% identity). OTU sequences were subsequently aligned using PyNAST (PyNAST: python nearest alignment space termination), after which taxonomy was assigned at 97% similarity against the SILVA SSURef database release 111. Alpha diversity estimates and beta diversity were calculated using QIIME and principal coordinate analysis (PCoA) plots were created in EMPeror (version 0.9.3-dev; Emperor) and used to visualize differences in beta diversity based on UniFrac distances. Finally, the Galaxy tool for determining the linear discriminant analysis (LDA) effect size (LEfSe) was used to identify taxa associated with particular interventions [24].

### Cecum short-chain fatty acids—GC-MS

Between 50–100 mg of cecal content from each animal were analyzed for SCFA composition as per the method described in Patterson et al. [25]. Briefly, a predetermined quantity of sample was vortexed with 1 ml of HPLC-grade water and allowed to incubate at room temperature for 10 min prior to centrifugation at 10,000 × *g* for 5 min. The supernatant was withdrawn and 3 · 0 mM of the internal standard, 2-ethylbutyric acid (Sigma Aldrich, Wicklow) was added. Samples were passed through a 0 · 22 μm filter and transferred to clean vials. Acetic, propionic, n-butyric, and butyric acids (Sigma Aldrich, Wicklow) were used as standards. Samples were then analyzed on a Varian 3500 GC flame ionization system, fitted with a Nukol-FFAP column (30 × 0 · 32 × 0 · 25 mm; Sigma) under the conditions previously reported. SCFA quantities are expressed as micromolar per gram cecum content.

### Fecal metabolome—^1^H-NMR spectroscopy

Briefly, fecal samples were broken down under liquid nitrogen by pestle and mortar, and ~40 mg of ground feces was weighted and transferred to microcentrifuge tube. The sample was suspended in 500 μl of HPLC-grade water by vortex mixing for 5 min at room temperature, followed by 30-min shaking at 4 °C. The suspension was then centrifuged at 22,400 × *g* for 30 min, at 4 °C. Supernatant was transferred to a new microcentrifuge tube and this was centrifuged again under the same conditions. Potassium buffer and D_2_O were added to the supernatant and the mixture was transferred to a clean NMR tube and analyzed on a 700 MHz Bruker NMR.

The resulting ^1^H-NMR spectra were processed and analyzed using BAYESIL [26], a fully-automated NMR spectral profiling program. A reference metabolite library was generated in the Chenomx NMR Suite Professional software package version 7.0 (Chenomx Inc., Edmonton, AB). The fits of a subset of the spectra were then verified by comparison with Chenomx.

### Serum metabolome—direct flow injection and LC-MS/MS

The serum collected as described previously was analyzed using the Biocrates AbsoluteIDQ p180 Kit (BIOCRATES Life Sciences AG, Austria), as described previously [27]. Following extraction and derivatization, analytes present in the samples were detected and quantified on an ABI 4000 Q-Trap mass spectrometer (MDS Sciex) run in conjunction with a reverse-phase HPLC-column. The analysis revealed levels of a range of specific amino acids, biogenic amines (BA), acylcarnitines (AC), lysophosphotidylcholines (lysoPC), phosphotidylcholines (PC), sphingomyelins (SM), and hexoses.

### Statistical analysis

Biochemical and biometric data were analyzed by one-way ANOVA against the HFC, performed in PASW Statistics v18.0 (IBM Corp., Chicago, IL, USA). Finally, linear regression was performed in Prism 5 (GraphPad Software Inc., CA USA). 16S compositional sequencing and metabolomics data were deemed to be non-normal and, as a result, non-parametric analyses were performed. All groups were analyzed by Kruskal-Wallis, followed by 2-tailed Mann–Whitney *U* pairwise comparisons, solely between HFC and each other group.

MetaboAnalyst metabolomics analysis suite [28–30] was utilized for normalization and multivariate analyses of data. PERMANOVA analyses of all groups together and direct comparisons of each treatment group against the HFC were performed using Adonis [Vegan] [31] on Bray-Curtis index of dissimilarity PCoA plot. Metabolomic and microbiome data were Pareto scaled prior to correlation analysis and plotting, while data were also surveyed, and samples which did not have the complete paired microbiome and relevant metabolome datasets were removed prior to Procrustes and coinertia analysis. For all analyses, a *p* value of less than 0.05 was considered to be statistically significant.

## Results

### Biochemical and biometric analysis

**Fig. 1:**
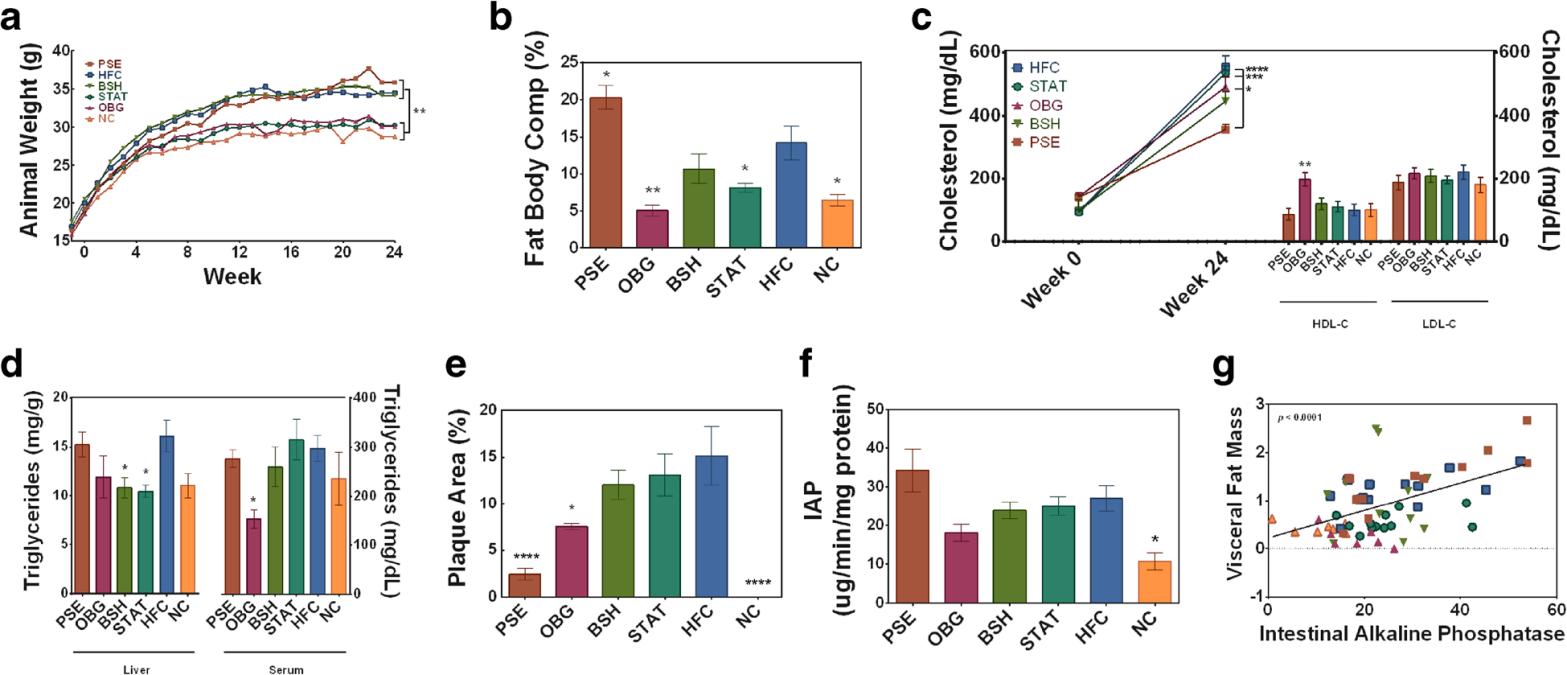
Effect of cardiovascular disease interventions on adiposity, cholesterol and lipid profile, atherogenesis, and inflammation. **a** Animal weight gain over the 24-week intervention period. **b** Percentage fat of animals prior to cull. **c** Serum total and HDL and LDL cholesterol evolution over 24-week period. **d** Liver and serum triglyceride levels following intervention. **e** Aortic plaque as percentage of total area. **f** Intestinal alkaline phosphatase (IAP) activity in jejunum tissue. **g** Positive correlation between visceral fat mass and IAP activity, with confidence bands displayed by dashed lines. *(*p* < 0.05), **(*p* < 0.01), and ***(*p* < 0.001) represent significant differences when compared against HFC in one-way ANOVA. Plots depict significant differences and plots depict individual replicates with mean and SEM

Animal body weights stratified the six groups into two significantly separate clusters (*p* < 0.01); the high-weight cluster which consists of PSE, HFC and BSH, and a low-weight cluster consisting of STAT, OBG, and NC—named in order of descending mean body weight (Fig. 1a). Both OBG (*p* < 0.01) and STAT (*p* < 0.05) mice presented with reduced percentage body fat when compared to the HFC (Fig. 1b). Conversely, PSE animals displayed a significant elevation in percentage body fat even when compared to the HFC (*p* < 0.05; Fig. 1b). PSE mice presented with significantly reduced total cholesterol when compared to the HFC (*p* < 0.05; Fig. 1c). Intervention with OBG increased HDL-C by ~100% when compared to the HFC (*p* < 0.01; Fig. 1c), while no effect on LDL-C was observed in any of the groups. Both OBG and STAT intervention significantly reduced liver weights when compared to HFC (*p* < 0.05; data not shown), while BSH and STAT reduced liver triglyceride levels (*p* < 0.05; Fig. 1d). Circulating triglyceride levels were found to be reduced only in OBG treated animals (*p* < 0.05 Fig. 1d).

High-fat feeding significantly increased the expression of IAP in the jejunum of all groups, bar OBG-fed mice, when compared to NC mice (*p* < 0.05; Fig. 1f). Furthermore, PSE mice were found to express the highest levels of the enzyme, ~35 μg/min/mg proteins, which also reached higher significance (*p* < 0.01).

### Atherosclerotic plaque scoring

Intervention with PSE offered substantial protection against the lipid-driven atherogenesis associated with the apo-E^−/−^ model, reducing total aortic plaque percentage from 15.2% (HFC) to 2.4% (*p* < 0.001; Fig. 1e). While OBG intervention reduced aortic plaque percentage to 7.4% (*p* < 0.05), neither of the other two interventions significantly attenuated plaque formation. The NC-fed group acted as a negative control for the function of the model, demonstrating no plaque whatsoever.

### Cecum microbiome compositional sequencing

**Fig. 2:**
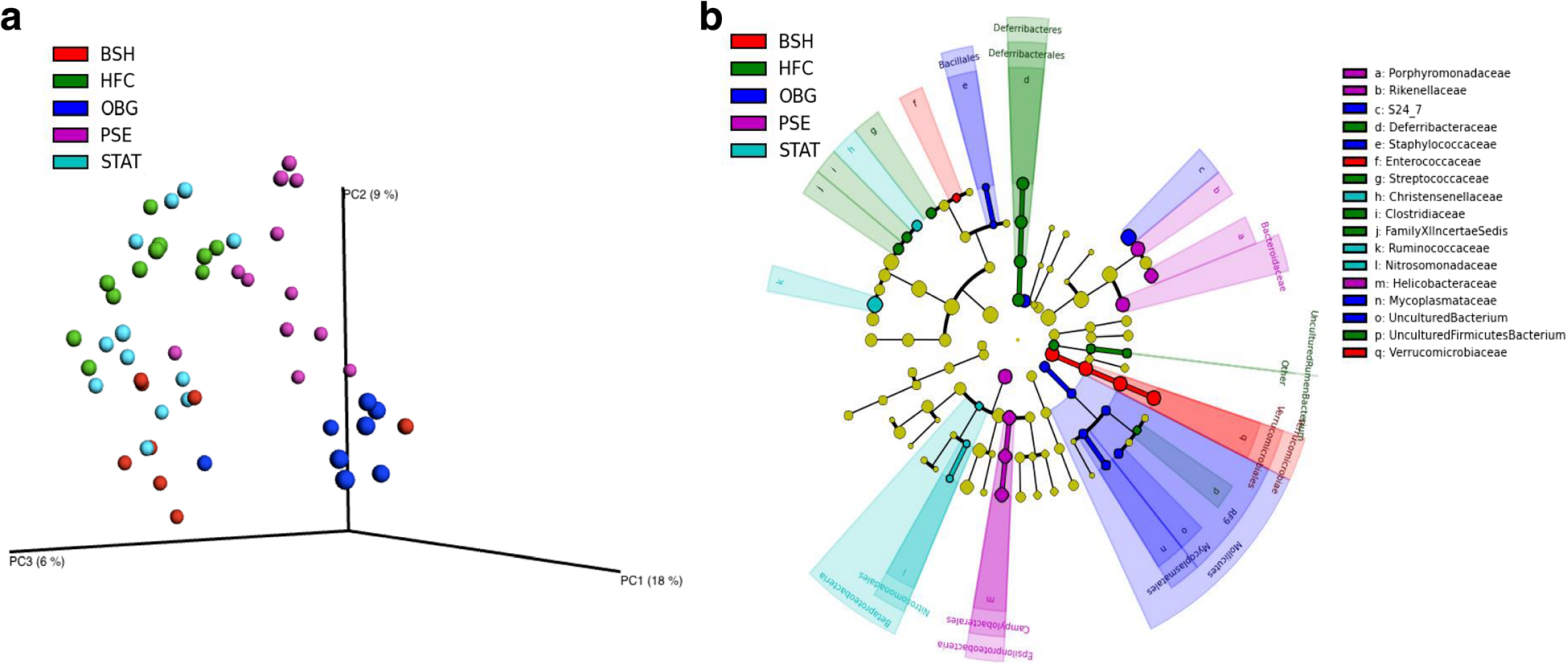
Effect of cardiovascular disease interventions on the microbiome composition. Unweighted unifrac principle coordinate analysis (PCoA) plots (**a**), with linear discriminant analysis (LDA) effect size (LEfSe) representation of taxa associated with HFC and each intervention (**b**)

NC animals scored higher than all other groups in each diversity metric, bar Simpson—in which NC was solely significantly higher than BSH (*p* < 0.05; Additional file 1: Table S1). OBG and HFC displayed similar diversities; while PSE, BSH and STAT animals had further reduced diversities compared to HFC for phylogenetic diversity whole tree and observed species (*p* < 0.05). Interestingly, there was a significant dissimilarity between groups overall (*p* < 0.01) and each intervention apart from STAT appeared to shift the microbiome β-diversity to such an extent that they clustered away from that of the HFC microbiome (Fig. 2a). However, PERMANOVA analysis of Bray-Curtis index of dissimilarity revealed that solely PSE clustered away from HFC significantly (*p* < 0.05), while BSH was approaching significance (*p* = 0.07). Fig. 2b depicts the taxa most associated with each of the interventions and HFC, as determined by LEfSe.

Phylum-relative abundance analysis revealed a significant increase in OBG (*p* < 0.01) and decrease in STAT (*p* < 0.05) *Verrucomicrobia* levels when compared to HFC (Additional file 1: Figure S1). *Proteobacteria* were found to be elevated in the PSE animals when compared to HFC (*p* < 0.01). This increase can be explained at the genus level primarily by a significant increase in *Helicobacter* (*p* < 0.01) and *Bilophila* (*p* < 0.05; Additional file 1: Figure S3). Finally, *Deferribacteres* were recorded at 0.9% relative abundance in HFC; while not one NC animal harbored the phylum (*p* = 0.056), and only one animal of each PSE (*p* < 0.05), OBG (*p* = 0.091), BSH (*p* = 0.075) and STAT (*p* < 0.05) presented with the taxon.

At family level, a significant reduction in *Streptococcaceae* was recorded in PSE (*p* < 0.01), OBG (*p* < 0.01), BSH (*p* < 0.05) and NC (*p* < 0.01). *Peptostreptococcaceae* (*p* < 0.05) and *Christenellaceae* (*p* < 0.01) relative abundances were reduced significantly in OBG and NC animals (Additional file 1: Figure S2). *Desulfovibrionaceae* were elevated in BSH animals (*p* < 0.05), while the PSE group displayed raised *Bacteroidaceae* (*p* < 0.01).

### Hosted cecum and fecal metabolome

**Fig. 3:**
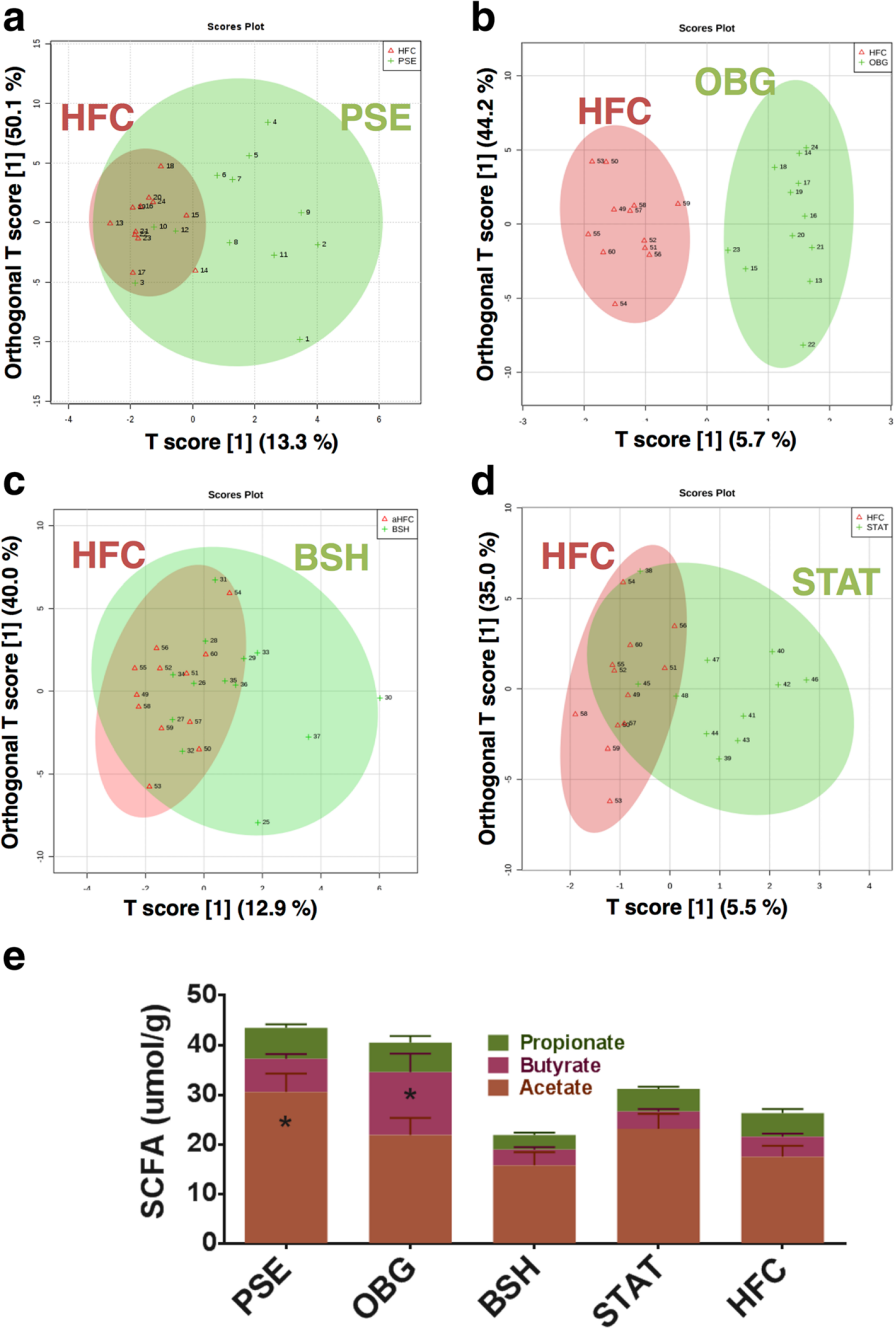
Effect of cardiovascular disease interventions on the hosted cecal and fecal metabolome. Fecal metabolome OPLS-DA: **a** PSE (*green*) vs. HFC (*red*). **b** OBG (*green*) vs. HFC (*red*). **c** BSH (*red*) vs. HFC (*green*). **d** STAT (*green*) vs. HFC (*red*). **e** Ceacum short-chain fatty acids (SCFA): Acetate (*brown*), n-Butyrate (*red*) and Propionate (*green*) were quantified in cecal content extracts by GC-MS

As anticipated, OBG mice displayed a SCFA profile most similar to that of NC, with significantly increased n-butyrate levels compared to HFC (*p* < 0.05; Fig. 3e). Interestingly, however, PSE proved capable of influencing acetate production most significantly when compared to HFC (*p* < 0.05; Fig. 3e), while BSH and STAT treatment appeared to have no detectable effect on these bacterial fermentation products.

Univariate analysis of log-transformed mouse fecal water metabolome data revealed a couple of interesting functional fluctuations, which may have implications for host cardiometabolic health. Intervention with PSE resulted in significantly reduced levels of trimethylamine (TMA) and isovalerate (*p* < 0.05). Supervised multivariate analysis in the form of partial least squares discriminant analysis (PLS-DA) and orthogonal PLS-DA (OPLS-DA) was carried out. Solely OBG intervention altered the fecal metabolome to cause clear cluster separation away from HFC in OPLS-DA plots (Fig. 3b). PLS-DA provided lists of metabolites that were found to be of most importance in explaining differences between each intervention and the HFC. PSE PLS-DA shifts were most strongly explained by reduced levels of isovalerate (*variable importance in projection [VIP] score* = 2.8) and TMA (*VIP score* = 2.2; Additional file 1: Figure S4A). OBG-treated mice were associated with formate (*VIP score* = 2.5) and lactate (*VIP score* = 2.1; Additional file 1: Figure S4B). In addition, BSH intervention was associated with reduced TMA (*VIP score* = 3.1) and elevated formate (*VIP score* = 3.0; Additional file 1: Figure S4C), while STAT also appeared to be associated most closely with formate (*VIP score* = 2.2; Additional file 1: Figure S4D). Finally, when the microbiome compositional PCoA matrix was compared with that of the fecal metabolome for each sample, we found no significant correlation in their grouping dissimilarity (Mantel statistic *r* = 0.004585; *p* > 0.05).

### Host serum metabolome

**Fig. 4:**
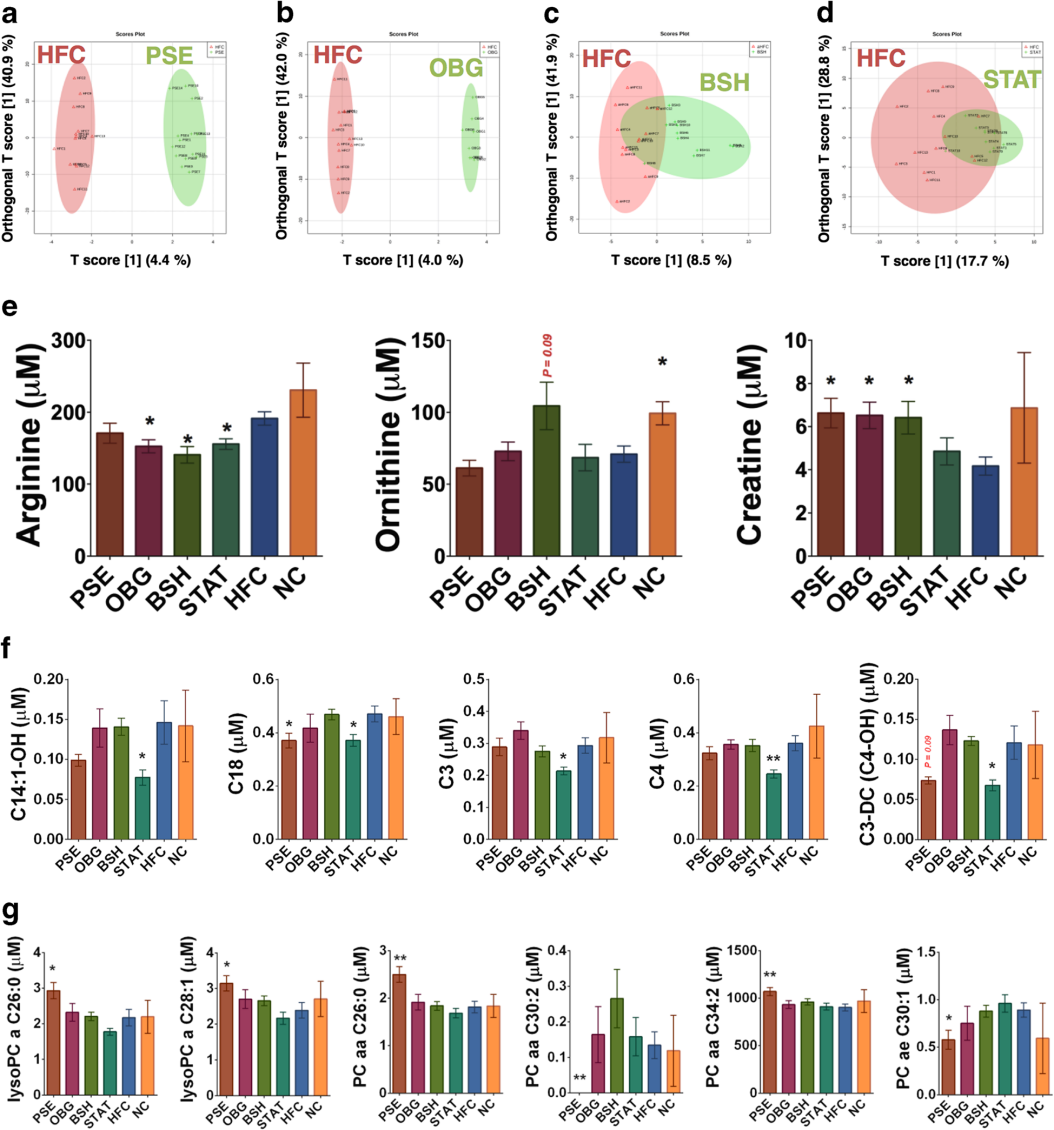
Effect of cardiovascular disease interventions on the host serum metabolome. Serum metabolome OPLS-DA: **a** PSE (*green*) vs. HFC (*red*). **b** OBG (*green*) vs. HFC (*red*). **c** BSH (*red*) vs. HFC (*green*). **d** STAT (*green*) vs. HFC (*red*). Significant differences in **e** amino acids and biogenic amines, **f** acylcarnatines, and **g** phosphotidylcholines in serum from each group, as analyzed by DI and LC-MS/MS. *(*p* < 0.05) and **(*p* < 0.01) represent significant differences when compared against HFC in one-way ANOVA. Plots depict individual replicates with mean and SEM

Analysis of the host serum lipid and AA metabolome revealed several clear functional shifts in response to the cardiovascular disease interventions, when compared to the HFC. OPLS-DA plots depict clear cluster separation of PSE (Fig. 4a) and OBG (Fig. 4b) away from the HFC. PLS-DA important feature plots identified reduced PC aa C30:2 as a very strong feature of the PSE mouse metabolome (*VIP score* = 10.6; Additional file 1: Figure S4A). C3-DC(C6-OH) was found to be of most importance in explaining OBG serum metabolome PLS-DA shifts (*VIP score* = 4.0; Additional file 1: Figure S4B). Finally, while kynurenine was deemed to be the most important feature in both BSH (*VIP score* = 7.0; Additional file 1: Figure S4C) and STAT (*VIP score* = 3.9; Additional file 1: Figure S4D), the relationship was negative in the latter. Interestingly, comparison of the microbiome compositional PCoA matrix with that of the fecal metabolome for each sample revealed a significant correlation in group dissimilarity (Mantel statistic *r* = 0.5013; *p* < 0.05).

In terms of specific amino acids, it was found that OBG, BSH, and STAT all significantly reduced arginine levels (*p* < 0.05), while NC increased ornithine (*p* < 0.05) and BSH trended towards an increase (*p* = 0.09; Fig. 4e). The biogenic amine creatine was significantly elevated by PSE, OBG, and BSH interventions (*p* < 0.05; Fig. 4e). No significant alterations to total amino acids, branched-chain amino acids or total biogenic amines were observed for any of the interventions or NC. In terms of AC, STAT appeared to have a relatively profound effect (Fig. 4f), significantly reducing C14:1-OH (i.e., hydroxytetradecenoylcarnitine; *p* < 0.05), C18 (i.e., octadecanoylcarnitine; *p* < 0.05), C3 (i.e., propionylcarnitine; *p* < 0.05), C4 (i.e., butyrylcarnitine; *p* < 0.01), and C3-DC(C4-OH) (i.e., malonylcarnitine+3-hydroxybutyrylcarnitine; *p* < 0.05). PSE was the only other intervention to impact on AC-metabolism, also reducing C18 significantly (*p* < 0.05) and trending towards a decrease in C3-DC(C4-OH) (*p* < 0.09). Finally, solely PSE intervention also demonstrated an impact on lysoPC and PC (Fig. 4g). The sterol intervention significantly increased lysoPC a C26:0 (*p* < 0.05), lysoPC a C28:1 (*p* < 0.05), PC aa C26:0 (*p* < 0.01), and PC aa C34:2 (*p* < 0.01), while decreasing PC ae C30:1 (*p* < 0.05) and abolishing PC aa C30:2 levels entirely (*p* < 0.01).

## Discussion

Although for many years the gut microbiome received little or no recognition for its contribution to the efficacy of certain functional food ingredients, we are now beginning to appreciate its true influence [32]. Furthermore, due to the broad metabolic capabilities of this microbial organ, it is likely that gut bacteria could manipulate a portion of the multitude of functional ingredients and drugs which are currently approved by regulatory authorities but lack a well-defined mechanism of action [33]. In this study, we aimed to characterize the effects of several commonly consumed nutritional cardiovascular disease interventions currently available commercially, as well as a widely prescribed statin, on the host and hosted metabolome through targeted and non-targeted metabolomic approaches, while also investigating the effects on the microbiome composition through 16S compositional sequencing.

### PSE

PSE-intervened mice displayed perhaps the most interesting physiological phenotype, with reduced cholesterol and aortic plaque, but the highest body weight and adiposity. The near complete eradication of aortic plaque was also coupled with a significant decrease in fecal TMA. Microbiome-derived TMA is known to pass into host circulation and travel to hepatocytes, in which flavin-containing monooxygenase-3 further metabolizes the compound to trimethylamine-*N*-oxide (TMAO) [2]—a process deemed to be highly correlated with atherogenesis [34, 3]. In addition, we observed several fascinating compositional microbiota fluctuations as a result of the PSE intervention. These animals presented with an elevated *Helicobacter* relative abundance, which was raised to 4.5%, while the genus was not detected at significant levels in any other group. Although colonization with *Helicobacter* can often remain harmless, the LPS and other antigens associated with the genus have shown the potential to induce a low-level systemic and vascular inflammatory tone in the host which may exacerbate atherogenesis [35]. Moreover, current infections with *Helicobacter pylori* have been associated with an altered, atherogenic lipid profile [36]. Increased LPS levels in host circulation characterize the low-level chronic inflammation, termed metabolic endotoxaemia, which is associated with high fat-induced metabolic dysfunction [37]. This scenario is thought to occur through increased intestinal permeability, which allows inflammatory bacterial fragments from the intestinal microbiota, such as LPS, to pass into circulation [5]. Bates et al. [38] demonstrated that LPS exposure triggered an increase in countervailing IAP expression, as the enzyme acts to detoxify the contaminant by dephosphorylation of the highly inflammatory lipid A moiety. All high fat-fed groups in this study, bar OBG, presented with elevated IAP levels when compared to NC-fed mice. This coincides with elevated *Proteobacteria*-relative abundances and visceral fat mass levels in these groups—effects from which OBG mice appeared to be afforded protection. The present study is possibly the longest run high fat-feeding trial available to report IAP levels in a mouse model, and thus, we propose that animals experiencing the greatest level of chronic endotoxemia may require augmentation of IAP expression to prevent low-level septicemia during high fat consumption.

PSE mice also displayed significantly increased percentage body fat, ~20% compared to ~15% in HFC mice. To date, there has been just one study reporting significant weight gain in apo-E-deficient mice following consumption of a phytosterol extract [39]. *Erysipelotrichaceae*, a family of bacteria previously associated with high fat consumption in murine models [40] and obesity in human cohorts [41], were found to be elevated in PSE when compared to HFC. Although each of the interventions appeared to stimulate *Erysipelotrichaceae* levels, PSE demonstrated the greatest propensity for this. This is peculiar, as it contradicts a report of Martínez et al*.*, in which a PSE diet (5% *w*/*w*) was found to decrease *Erysipelotrichaceae* abundance in hamsters [8]. However, PSE animals also presented with elevated cecum acetate production and it seems likely that a member of the *Bacteroidaceae* family (such as *Bacteroides* or *Parabacteroides* [42]) may have been responsible for the significant increase in acetate production in PSE animals. SCFA are key microbial fermentation metabolites which, through activation of G-protein-coupled receptors (GPCR)-41 and 43, can have important implications for host metabolic function [43]. However, the work of Turnbaugh et al. [44] a decade ago highlighted an increased energy harvesting capacity of the obesity-associated microbiota, which was attributed most significantly to acetate production. Taken together, these data may indicate a causative role of the microbiome in the degree of adiposity of PSE-treated animals.

Interestingly, while levels of the genus *Roseburia*—a group of microbes which express butyryl-coenzyme A, an enzyme involved in the conversion of acetate to butyrate—were increased >250% in the PSE mice compared to HFC, the ratio of acetate:butyrate remained high. *Roseburia* spp. have also been proven to metabolize linoleic acid into two precursors of the c9, t11-conjugated linoleic acid (CLA) isomer [45], a bacterial metabolite which has shown potential in the attenuation of inflammatory disorders such as cardiovascular disease [46]. Finally, PSE mice were again found to be unique in that they displayed an altered PC and LysoPC profile. This is certainly interesting, as PSE are known to interact with PC, while directly competing with cholesterol in the formation of biliary micelles [47].

### OBG

Butyrate levels were found to be elevated in cecal content of OBG-treated mice, suggesting a prebiotic effect. In contrast to acetate, butyrate and propionate have previously been found to protect against diet-induced obesity in a murine model [48], which may explain the body weight and percentage fat displayed by OBG-intervened mice. In addition, this increase in butyrate could also explain the prevention of IAP expression increase observed in all other high fat-fed groups, as the SCFA has been shown to have important implications for tight-junction function in the gut barrier [43, 49]. As anticipated, OBG intervention again appeared to exhibit prebiotic effects on the cecal microbiota through stimulation of *Verrucomicrobia* population expansion. *Akkermansia*, the sole identified gut microbiota member genus of the phylum *Verrucomicrobia* [50], has been shown to have major implications for host metabolic health, including metabolic endotoxaemia [51–53]. While prebiotic-mediated promotion of *Akkermansia muciniphila* levels has displayed great potential in stimulating gut peptide production [51], thereby improving tight junction function and insulin sensitivity [13], it is also a potent mucin degrader—a process in which sulfate is released as a by-product [54]. The elevated levels of *Akkermansia* in the OBG-treated animals therefore most likely explain the simultaneous expansion of sulfate-reducing *Desulfovibrio* populations, as has previously been observed in a healthy adolescent human cohort [55]. *Desulfovibrio* spp. display a relatively unique and potentially harmful metabolic profile, reducing sulfates to potentially carcinogenic sulfides [56] and catabolizing choline to potentially atherogenic TMA [57]. Interesting in this regard was the fact that OBG-intervened mice demonstrated the highest mean fecal TMA levels; however, this was not significantly higher than HFC animals. Despite this, OBG mice appeared to be protected from the high-fat/cholesterol-induced atherogenesis. Moreover, in the previous mentioned adolescent study, both *Akkermansia* and *Desulfovibrio* levels were associated with the healthy control cohort rather than the obese/overweight group [55].

### BSH

VSL#3 is a probiotic mix of bifidobacteria, lactobacilli, and streptococci cultures which has displayed BSH activity in vivo [58]. While the BSH-active *Lactobacillus reuteri* investigated in this study was associated with increased *Verrucomicrobia* levels, when VSL#3 was applied to a murine model of colonic cancer the luminal and mucosal-adherent levels of the phylum were greatly reduced [59]. As with OBG intervention, this increase in *Verrucomicrobia* was again associated with an increase in *Desulfovibrionaceae*. However, the expansion of OBG *Desulfovibrionaceae* levels was solely the result of increased *Desulfovibrio*, in contrast with BSH animals there was no such effect on the TMA-producing genus—perhaps explaining the lack of increase in proatherogenic metabolites in these animals. In fact, the increase was due to raised levels of taurine-degrading bacterium, *Bilophila* [60]. High fat consumption has previously been shown to increase levels of the pathobiont *Bilophila wadsworthia* [61], although the same effect has also been achieved by increasing host tauro-conjugated bile acid levels. This makes it particularly interesting that the genus was increased in the BSH group in the present study, when compared to the control. A recent clinical study investigating the effects of daily consumption of BSH-active *Lb. reuteri* NCIMB 30242 in a delayed release capsule uncovered a significant and sustained increase in both glyco and tauro-conjugated bile acid levels over a 4-week intervention period in otherwise healthy, hypercholesterolemic subjects [62]. The implications of such a fluctuation to host cardiovascular disease risk are currently unclear; however, it may be important to consider how BSH intervention may affect populations of this potentially inflammatory pathobiont in the human intestinal microbiota.

### STAT

Simvastatin is an example of a cardiovascular disease drug which modulates gut microbiota composition [63] and whose efficacy appears to rely on a suitably responsive microbiome [64]. Simvastatin has previously been shown to boost *Lactobacillus* populations, while in the present study Atorvastatin showed little global effect (Fig. 2a). However, the STAT group was the only intervention group to display an increase in *Ruminococcus*-relative abundance (of ~50%) when compared to HFC-fed animals. Certain *Ruminococcus* spp. have been described as key-stone species in the catabolism of resistant starch [65] and mucin [66]; and therefore promote a diverse, saccharolytic microbiota which may have implications for host metabolic health. Despite having only a modest effect on microbiome composition and functionality, STAT appeared to shift the host serum AC composition, reducing the levels of several of these molecules which play central roles in lipid metabolism. In line with this, serum AC concentrations have been positively correlated with obesity and other metabolic dysfunctions [67]. Furthermore, several of the same AC which were reduced by STAT intervention have previously been positively associated with glucose intolerance in prediabetic and type-2 diabetic cohorts [68]. Intriguingly, STAT animal physiology was most similar to that of OBG animals—presenting with reduced liver weight, body weight, and a near significant reduction in percentage body fat—potentially as a direct result of these AC-modifying attributes. Despite all of these metabolism-linked alterations, no attenuation effect on atherogenic plaque development was noted following STAT intervention. This is not surprising given that previous work failed to demonstrate an ability of a Simvastatin-based intervention to prevent plaque development in the same murine model [69].

## Conclusions

This study has highlighted the impact of commonly consumed nutritional and pharmaceutical cardiovascular disease interventions on the gut microbiome. In particular, the data show disconnect between weight gain and adiposity versus atherogenic plaque development in PSE-intervened mice, which is intriguing. This observation coincided with reductions of proatherogenic and metabolism-modulating microbial metabolites in fecal water, and the greatest alterations in microbiome composition relative to the HFC. In addition, we demonstrate the impact of these alterations on the host serum metabolome, in the context of cardiometabolic function. Overall, OBG intervention caused the most favorable shifts in microbiome composition and functionality, which may have contributed to the healthy phenotype observed in this group. On balance, it appears that OBG may be the preferred dietary intervention for safe long-term maintenance of cardiovascular and metabolic health, potentiated by the microbiota. As novel therapeutics enter the market, it becomes increasingly important that we consider their effects on our microbial organ and disentangle direct drug actions from drug-microbiome mediated actions.

## Additional file

Additional file 1:
**Figure S1.** Phylum-relative abundances of intervention and control groups. (i) Phylum-relative abundances, with inset plots representing phyla in which a significant difference between HFC and another group has been identified. *(*p* < 0.05) and **(*p* < 0.01) represent significant differences recorded between HFC and one of the interventions, or NC. Plots depict individual replicates with mean and SEM. **Figure S2.** Family-relative abundances of intervention and control groups. *(*p* < 0.05) and **(*p* < 0.01) represent significant differences recorded between HFC and one of the interventions, or NC. Plots depict individual replicates with mean and SEM. **Figure S3.** Genus-relative abundances of intervention and control groups. Genus-relative abundances, with inset plots representing genera in which a significant difference between HFC and another group have been identified. *(*p* < 0.05) and **(*p* < 0.01) represent significant differences recorded between HFC and one of the interventions, or NC. Plots depict individual replicates with mean and SEM. **Figure S4.** Fecal metabolome important features associated with each cardiovascular disease intervention. The fecal metabolites found to be the most important in explaining shifts in PLS-DA for each intervention (PSE [A], OBG [B], BSH [C], and STAT [D]) when compared to the HFC. **Figure S5.** Serum metabolome important features associated with each cardiovascular disease intervention. The serum metabolites found to be most important in explaining shifts in PLS-DA for each intervention (PSE [A], OBG [B], BSH [C], and STAT [D]) when compared to the HFC. **Figure S6.** Correlation heatmap containing physiological, microbiome, and fecal metabolome data. **Figure S7.** Correlation coefficient analysis plots of primary physiological outcomes with microbiome and fecal metabolome. Several important metabolic markers are displayed with the microbial taxa and metabolites most correlated (Cholesterol [A], Plaque [B], TAG [C], and IAP [D]). *FDR-adjusted *p* < 0.05. **Table S1.** Alpha diversity of intervention and control groups. Means in a row with common superscripts do not differ (*p* ≥ 0.05). (DOCX 2462 kb)

## Abbreviations

AC: Acylcarnitines
BSH: Bile salt hydrolase
CLA: Conjugated linoleic acid
GLP-1: Glucagon-like peptide-1
GLP-1R: Glucagon-like peptide-1 receptor
GPCR: G-protein-coupled receptor
HDL-C: High-density lipoprotein cholesterol
HFC: High-fat/cholesterol control
IAP: Intestinal alkaline phosphatase
LDA: Linear discriminant analysis
LDL-C: Low-density lipoprotein cholesterol
LEfSe: Linear discriminant analysis effect size
lysoPC: Lysophosphotidylcholines
MRS: De Man Rugosa Sharp
NC: Normal chow
OBG: Oat β-glucan
OPLS-DA: Orthogonal partial least squares discriminant analysis
OTU: Operational taxonomic units
PC: Phosphotidylcholines
PCoA: Principal coordinate analysis
PFA: Paraformaldehyde
PLS-DA: Partial least squares discriminant analysis
PSE: Plant sterol ester
PYY: Peptide YY
SM: Sphingomyelins
STAT: Statin
TG: Triglycerides
TMA: Trimethylamine
TMAO: Trimethylamine-*N*-oxide
VIP: Variable importance in projection.
