# Supplementary material for: Microbiome and metabolome modifying effects of several cardiovascular disease interventions in apo-E−/− mice
Source: Microbiome. 2017 Mar 13;5:30. doi: 10.1186/s40168-017-0246-x (PMC5346842; doi:10.1186/s40168-017-0246-x)
Supplement: Additional file 1: — Figure S1. Phylum-relative abundances of intervention and control groups. (i) Phylum-relative abundances, with inset plots representing phyla in which a significant difference between HFC and another group has been identified. *(p < 0.05) and **(p < 0.01) represent significant differences recorded between HFC and one of the interventions, or NC. Plots depict individual replicates with mean and SEM. Figure S2. Family-relative abundances of intervention and control groups. *(p < 0.05) and **(p < 0.01) represent significant differences recorded between HFC and one of the interventions, or NC. Plots depict individual replicates with mean and SEM. Figure S3. Genus-relative abundances of intervention and control groups. Genus-relative abundances, with inset plots representing genera in which a significant difference between HFC and another group have been identified. *(p < 0.05) and **(p < 0.01) represent significant differences recorded between HFC and one of the interventions, or NC. Plots depict individual replicates with mean and SEM. Figure S4. Fecal metabolome important features associated with each cardiovascular disease intervention. The fecal metabolites found to be the most important in explaining shifts in PLS-DA for each intervention (PSE [A], OBG [B], BSH [C], and STAT [D]) when compared to the HFC. Figure S5. Serum metabolome important features associated with each cardiovascular disease intervention. The serum metabolites found to be most important in explaining shifts in PLS-DA for each intervention (PSE [A], OBG [B], BSH [C], and STAT [D]) when compared to the HFC. Figure S6. Correlation heatmap containing physiological, microbiome, and fecal metabolome data. Figure S7. Correlation coefficient analysis plots of primary physiological outcomes with microbiome and fecal metabolome. Several important metabolic markers are displayed with the microbial taxa and metabolites most correlated (Cholesterol [A], Plaque [B], TAG [C], and IAP [D]). *FDR-adjusted p < 0. [file 40168_2017_246_MOESM1_ESM.docx]

**ONLINE SUPPLEMENTAL MATERIAL**

**Supplementary Figure S1 – Phylum relative abundances of intervention and control groups.** *(i) Phylum relative abundances, with inset plots representing phyla in which a significant difference between HFC and another group has been identified. * (p < 0.05) and ** (p < 0.01) represent significant differences recorded between HFC and one of the interventions, or NC. Plots depict individual replicates with mean and SEM, and dashed lines depict the HFC mean for use as comparison.*

**Supplementary Figure S2 – Family relative abundances of intervention and control groups.** *Column A inset plots represent families that contain species capable of converting choline into pro-atherogenic TMA. Column B represents families containing species known to produce SCFA, which interact with host cardiometabolic health. Column C represents families containing other species of interest for host metabolic and immunological health. * (p < 0.05) and ** (p < 0.01) represent significant differences recorded between HFC and one of the interventions, or NC. Plots depict individual replicates with mean and SEM, and dashed lines depict the HFC mean for use as comparison.*

**Supplementary Figure S3 – Genus relative abundances of intervention and control groups.** *Genus relative abundances, with inset plots representing genera in which a significant difference between HFC and another group has been identified. * (p < 0.05) and ** (p < 0.01) represent significant differences recorded between HFC and one of the interventions, or NC. Plots depict individual replicates with mean and SEM, and dashed lines depict the HFC mean for use as comparison.*

**Supplementary Figure S4 – Faecal metabolome important features associated with each cardiovascular disease intervention.** *The faecal metabolites found to be most important in explaining shifts in PLS-DA for each intervention (PSE [****A****], OBG [****B****], BSH [****C****] and STAT [****D****]) when compared to the HFC.*

**Supplementary Figure S5 – Serum metabolome important features associated with each cardiovascular disease intervention.** *The serum metabolites found to be most important in explaining shifts in PLS-DA for each intervention (PSE [****A****], OBG [****B****], BSH [****C****] and STAT [****D****]) when compared to the HFC.*

**Supplementary Figure S6 – Correlation heatmap containing physiological, microbiome and faecal metabolome data.**

**Supplementary Figure S7 – Correlation coefficient analysis plots of primary physiological outcomes with microbiome and faecal metabolome.** *Several important metabolic markers are displayed with the microbial taxa and metabolites most correlated (Cholesterol [****A****], Plaque [****B****], TAG [****C****] and IAP [****D****]). * FDR adjusted p < 0.05.*

**Supplementary Table S1 –** **Alpha diversity of intervention and control groups.** *Means in a row with common superscripts do not differ (P ≥ 0.05).*

**SUPPLEMENTARY FIGURE S1**


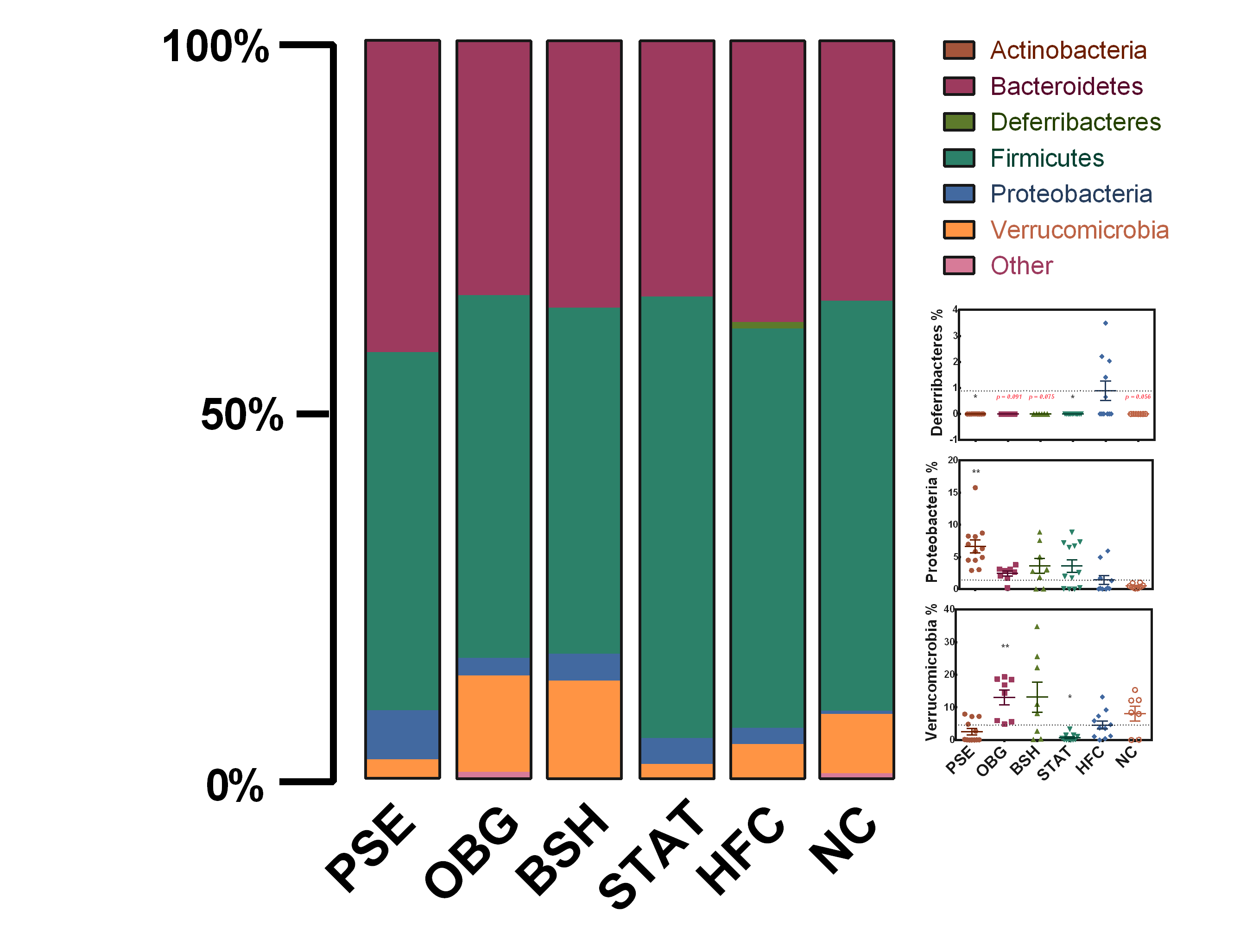


**SUPPLEMENTARY FIGURE S2**

*
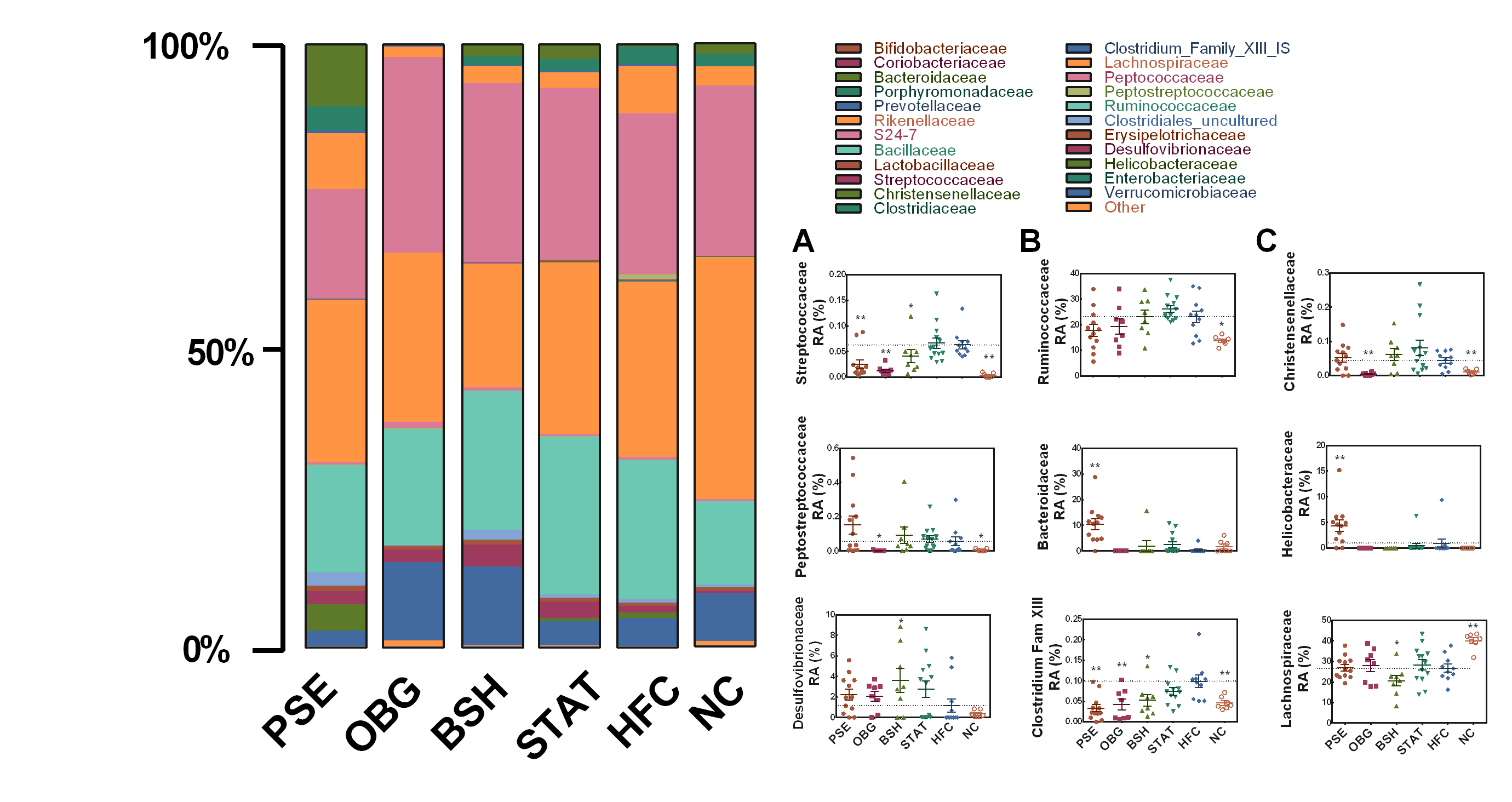
*

**SUPPLEMENTARY FIGURE S3**

**
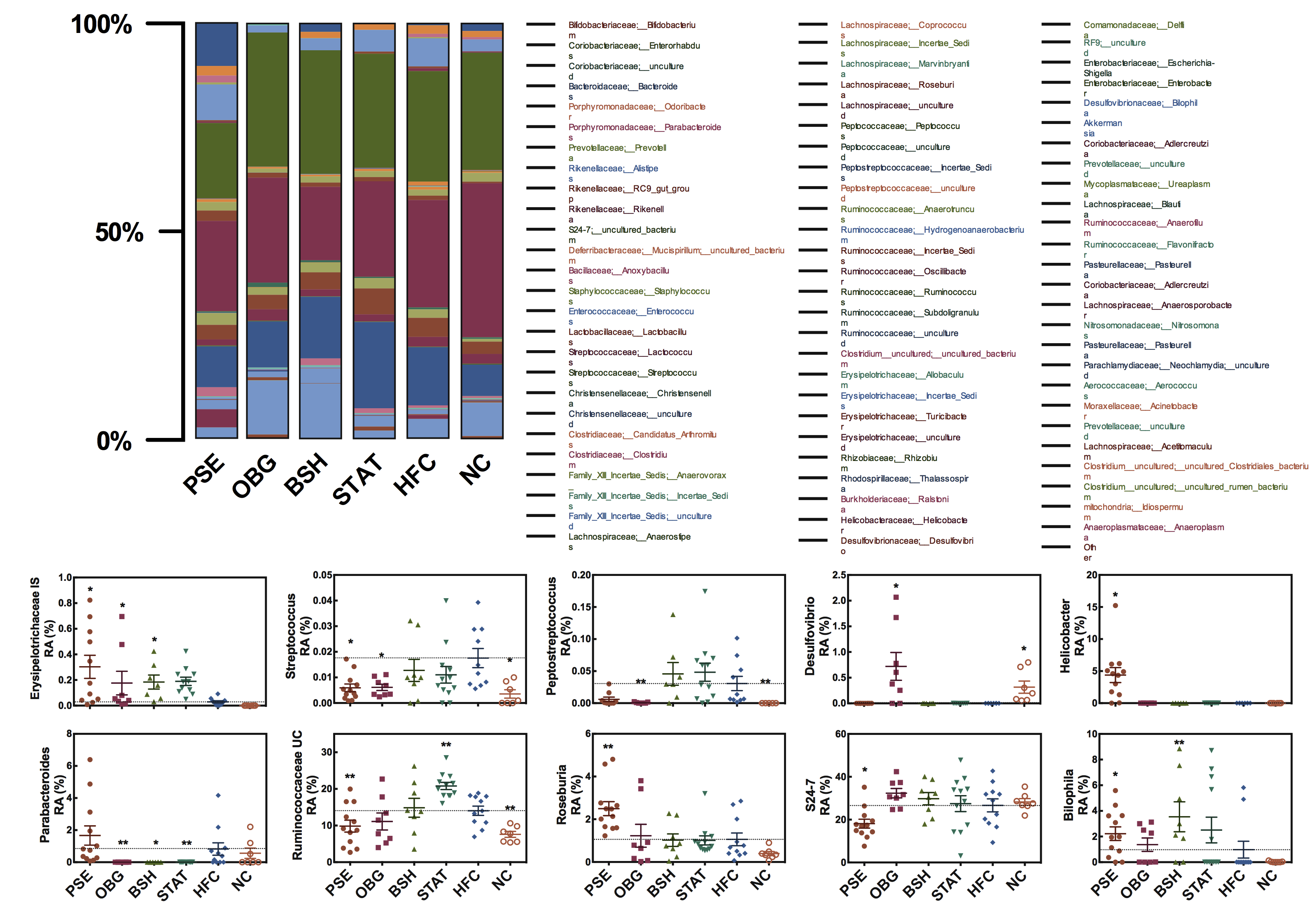
**

**SUPPLEMENTARY FIGURE S4**

**
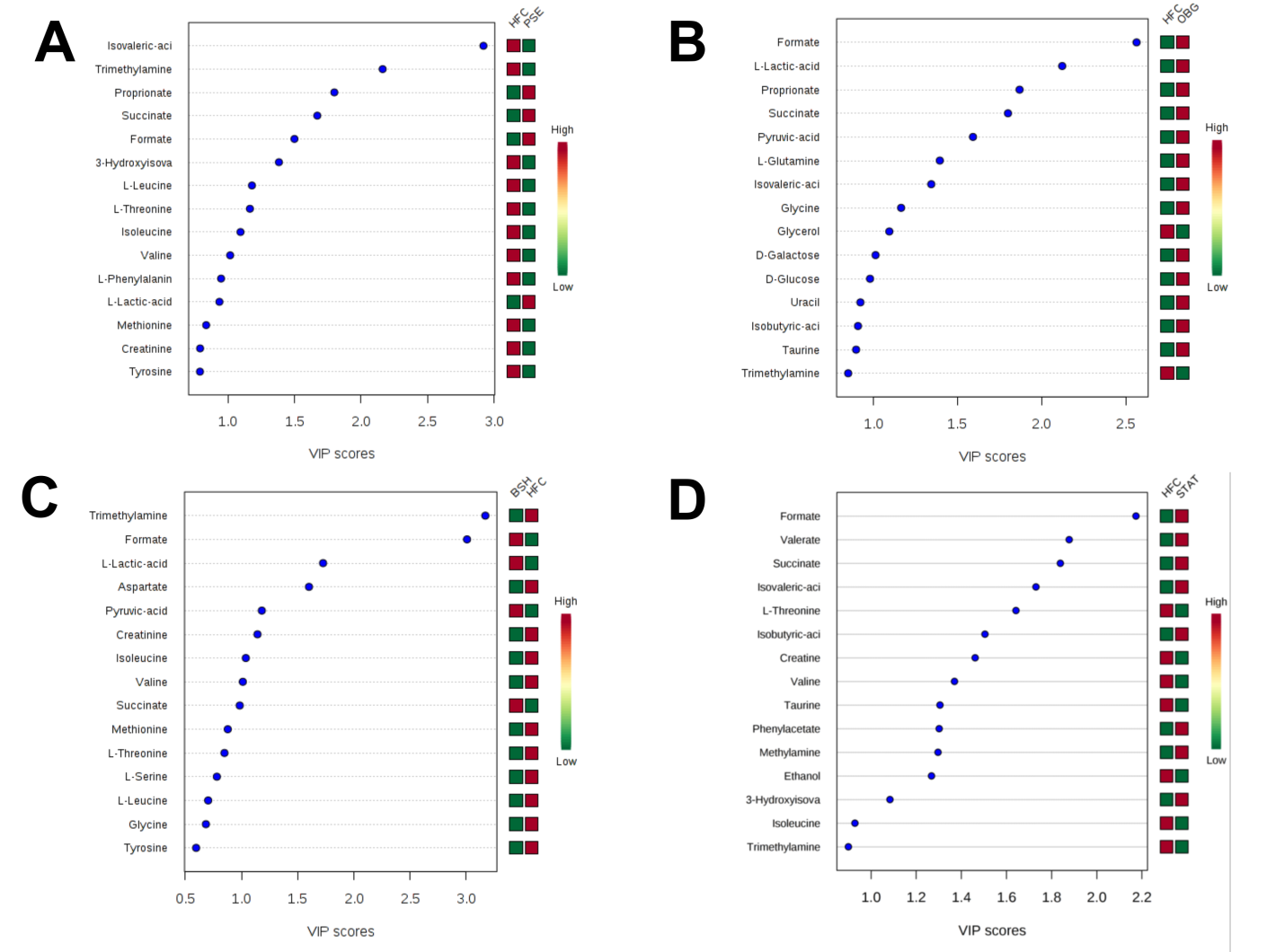
**

**SUPPLEMENTARY FIGURE S5**

**
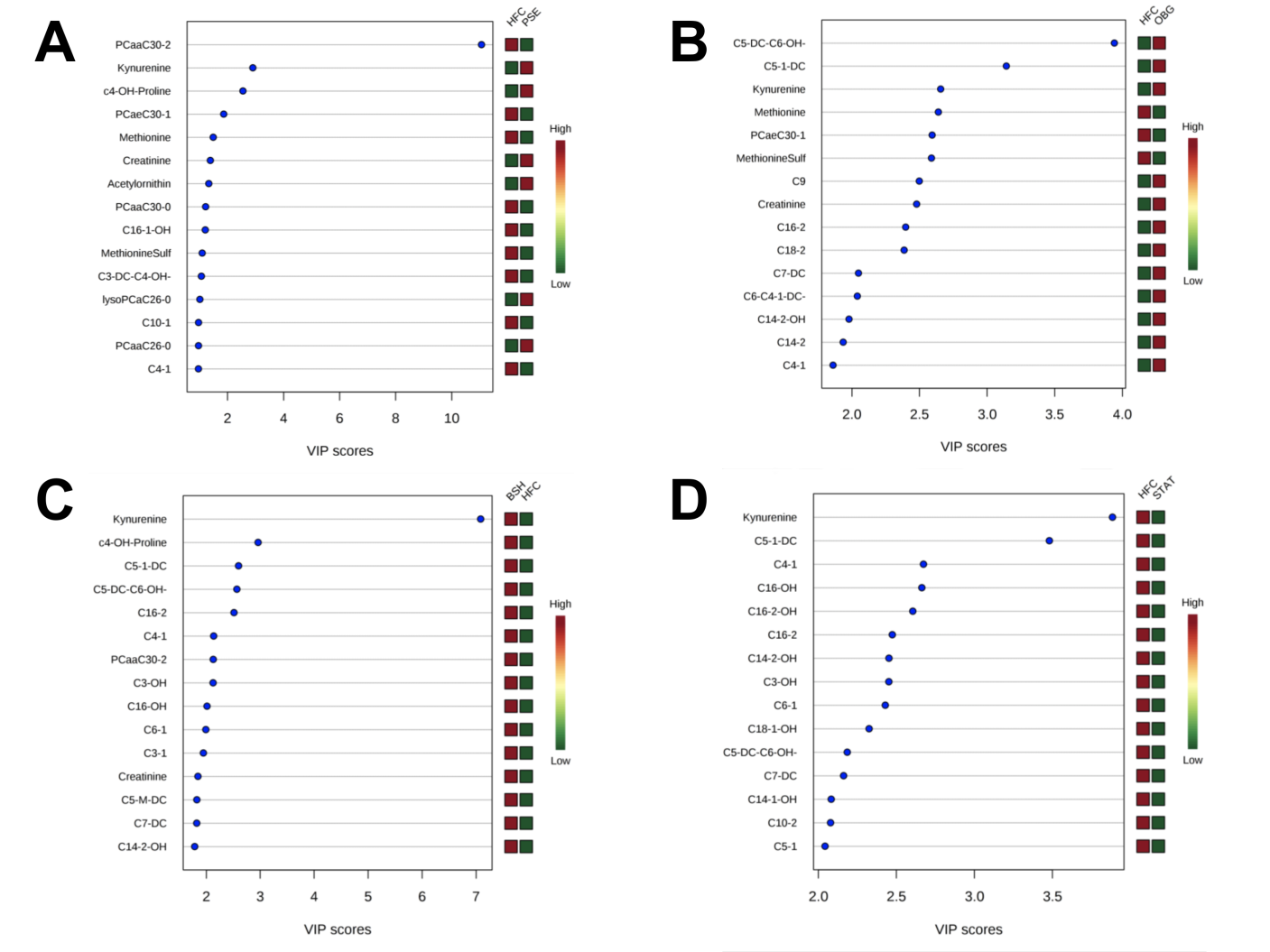
**

**SUPPLEMENTARY FIGURE S6**

**
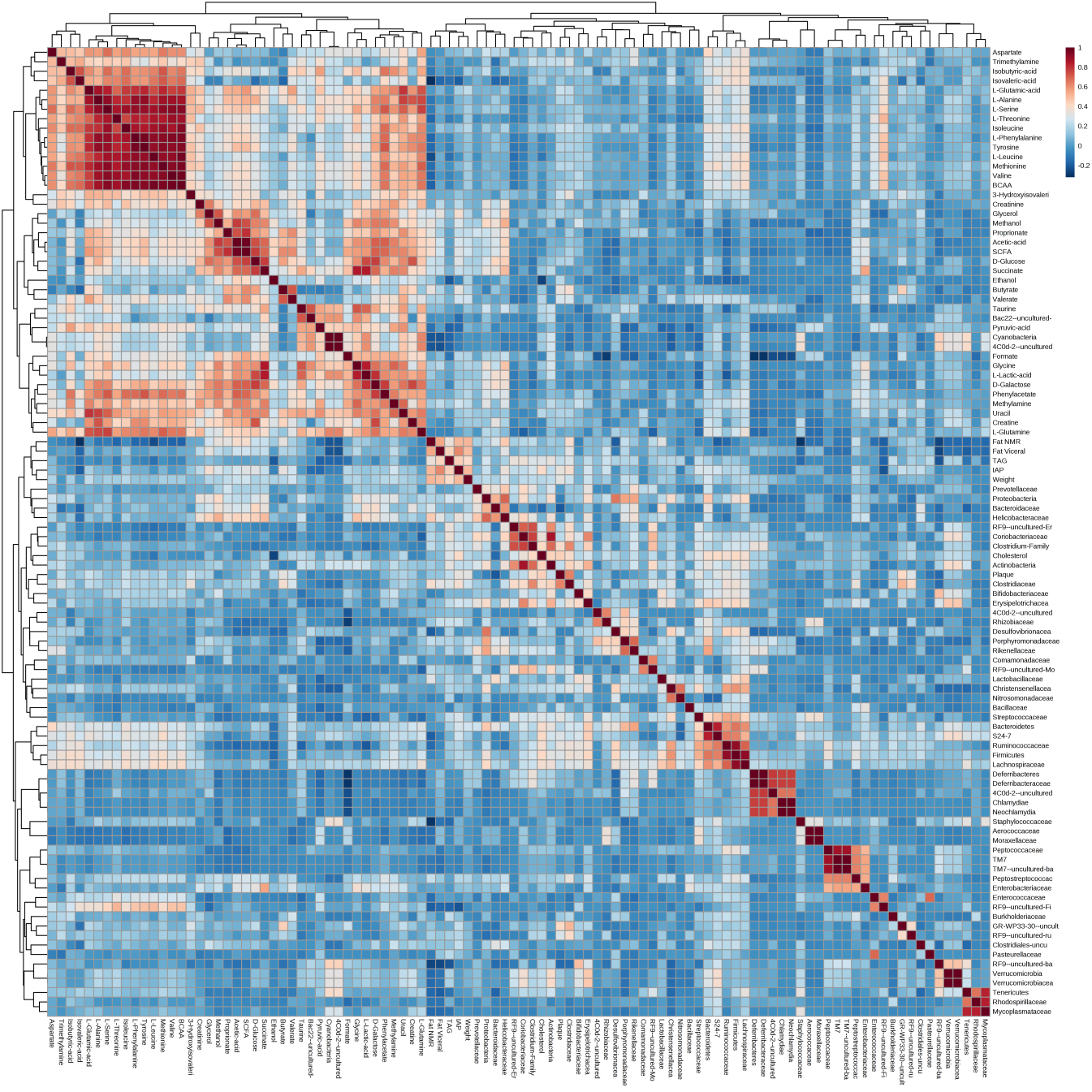
**

**SUPPLEMENTARY FIGURE S7**

**
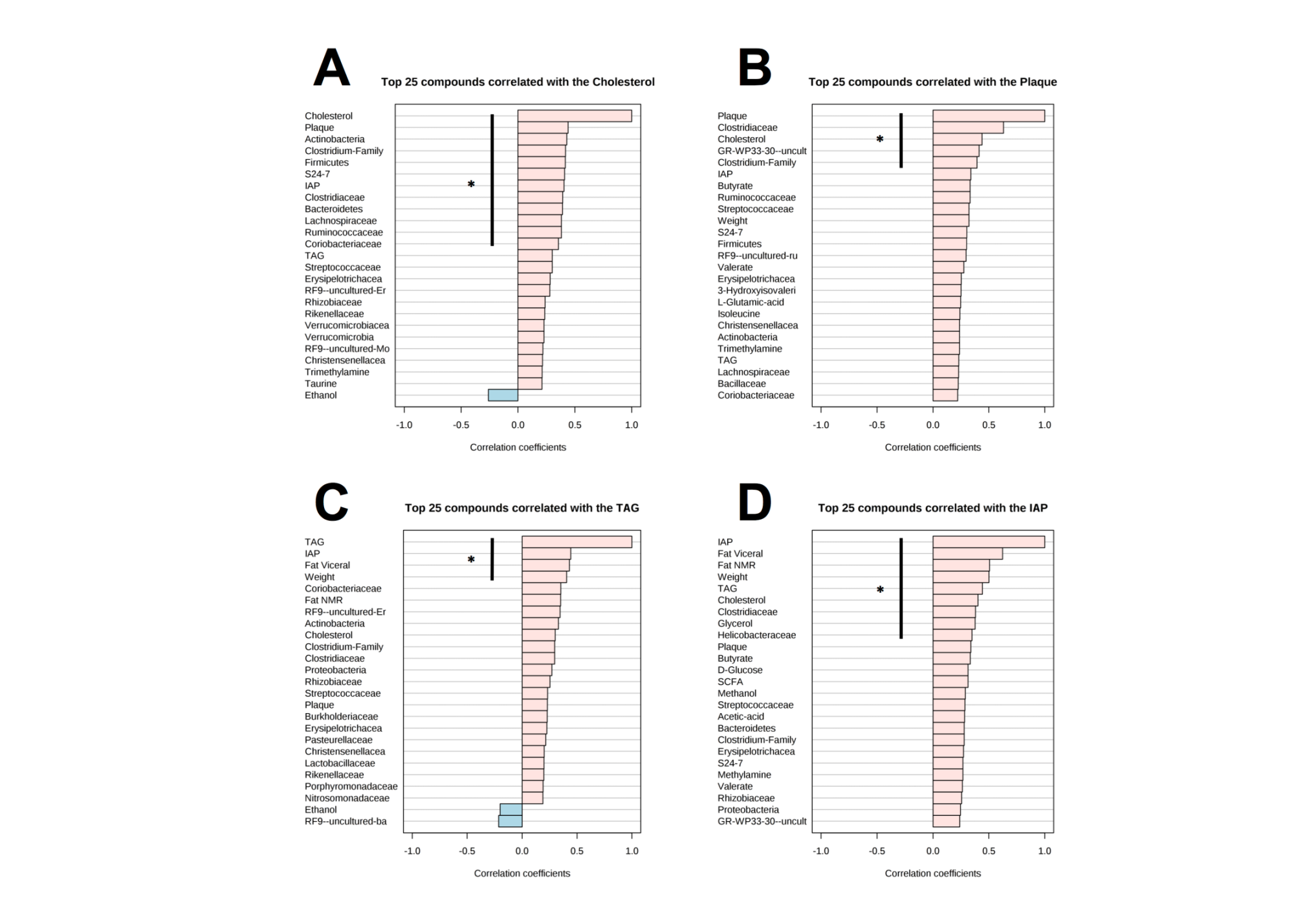
**

**SUPPLEMENTARY TABLE S1**

|  | **PSE** | **OBG** | **BSH** | **STAT** | **HFC** | **NC** |
| --- | --- | --- | --- | --- | --- | --- |
| **Chao1** | 276.75 ^bc^ | 299.85 ^bc^ | 266.90 ^c^ | 290.15 ^bc^ | 315.39 ^bc^ | 443.73 ^a^ |
| **Simpsons** | 0.95 ^ab^ | 0.95 ^ab^ | 0.93 ^b^ | 0.95 ^ab^ | 0.96 ^ab^ | 0.97 ^a^ |
| **Shannon** | 5.30 ^bc^ | 5.39 ^bc^ | 5.09 ^c^ | 5.31 ^bc^ | 5.62 ^bc^ | 6.13 ^a^ |
| **PD Whole Tree** | 13.96 ^d^ | 15.85 ^bc^ | 13.85 ^d^ | 14.53 ^cd^ | 16.39 ^b^ | 22.45 ^a^ |
| **Observed Species** | 230.33 ^cd^ | 259.88 ^bc^ | 218.50 ^d^ | 238.08 ^cd^ | 271.64 ^b^ | 397.14 ^a^ |
